# Improvement of arthroscopic surgical performance using a new wide-angle arthroscope in the surgical training

**DOI:** 10.1371/journal.pone.0203578

**Published:** 2019-03-11

**Authors:** Jae-Man Kwak, Erica Kholinne, Maulik Gandhi, Arnold Adikrishna, Hanpyo Hong, Yucheng Sun, Kyoung-Hwan Koh, In-Ho Jeon

**Affiliations:** 1 Department of Orthopedic Surgery, Asan Medical Center, College of Medicine, Ulsan University, Seoul, South Korea; 2 Department of Orthopedic Surgery, St. Carolus Hospital, Jakarta, Indonesia; 3 Upper Limb Department, Robert Jones & Agnes Hunt Hospital, Oswestry, England, United Kingdom; Universidade Federal Fluminense, BRAZIL

## Abstract

**Background:**

We have developed a new arthroscope with a field of view of 150°. This arthroscope requires less motion to maneuver and exhibits reduced optical error. It also improves how novices learn arthroscopy. We hypothesized that the surgical performance with this arthroscope is superior to that with a conventional arthroscope. This study tested the hypothesis by using motion analysis and a new validated parameter, “dimensionless squared jerk” (DSJ).

**Methods:**

We compared the surgical performance between the use of the wide-angle arthroscope and that of the conventional arthroscope among 14 novice orthopedic residents who performed 3 standardized tasks 3 times with each arthroscope. The tasks simulated the surgical skills in arthroscopic rotator cuff repair. The arthroscope motion was analyzed using an optical tracking system. The differences in performance parameters, such as the time taken to complete the tasks, average acceleration of the hands (m/s^2^), number of movements, and total path length (m) including DSJ between the 2 arthroscopes were investigated using paired t-tests.

**Results:**

All estimated values for the tasks using the 150° arthroscope were lower than those for the tasks using the 105° arthroscope. Statistically significant differences in performance between the 2 arthroscopes were observed only for DSJ (p = 0.014) and average acceleration (p = 0.039).

**Conclusions:**

DSJ and average acceleration are reliable parameters for representing hand-eye coordination. The surgical performance of novice arthroscopists was better with the new wide-angle arthroscope than with the conventional arthroscope.

## Introduction

Shoulder arthroscopy has evolved and has become an important method in minimally invasive surgery. As arthroscopic techniques have evolved, arthroscopy has become the gold standard method for the diagnosis and management of shoulder disorders [1–4]. However, several issues remain, particularly with respect to the long learning curve and the time required for training. Additionally, technical errors arise from the limitations of hand-eye coordination required for the use of arthroscopic systems, especially for novice arthroscopists. Several studies have shown that training using a dry or virtual simulator is effective and safe for overcoming these issues [5–7]. However, cost-effectiveness remains an issue in every training hospital.

On the basis of our experience, one of the main problems in arthroscopy is the limited field of view (FOV) of conventional arthroscopic systems. Several studies have reported that a limited arthroscopic FOV can make it difficult to identify entire anatomic structures, and this was often associated with poor hand-eye coordination during surgical triangulation and handling of the instrument [8–11]. We hypothesized that a wide-angle arthroscope with a higher FOV would result in better hand-eye coordination and would bring important benefits such as acceleration of learning, reduction of technical errors, and improvement in the long-term performance of surgical skills.

The aim of this study was to compare hand-eye coordination and the performance of surgical skills between the use of a newly developed wide-angle arthroscope (150° FOV) and that of the conventional arthroscope (105° FOV). We used motion analysis to evaluate the performance of novice arthroscopists. We assessed their hand-eye coordination by using the “dimensionless squared jerk” (DSJ)[12], which is widely accepted as an objective parameter of hand-eye coordination in the field of engineering.

## Materials and methods

All procedures performed in studies involving human participants were in accordance with the ethical standards of the institutional and/or national research committee and with the 1964 Helsinki Declaration and its later amendments or comparable ethical standards. Institutional Review Board Approval was obtained from Asan Medical Center prior to study (no. 2017–0292). Written Informed consent was obtained from all individual participants included in the study.

### Participants

A total of 14 right-handed orthopedic residents with no prior arthroscopic surgical training were recruited by announcement for open recruitment in Asan Medical Center during May 2017.

### Arthroscopic systems

**Fig 1:**
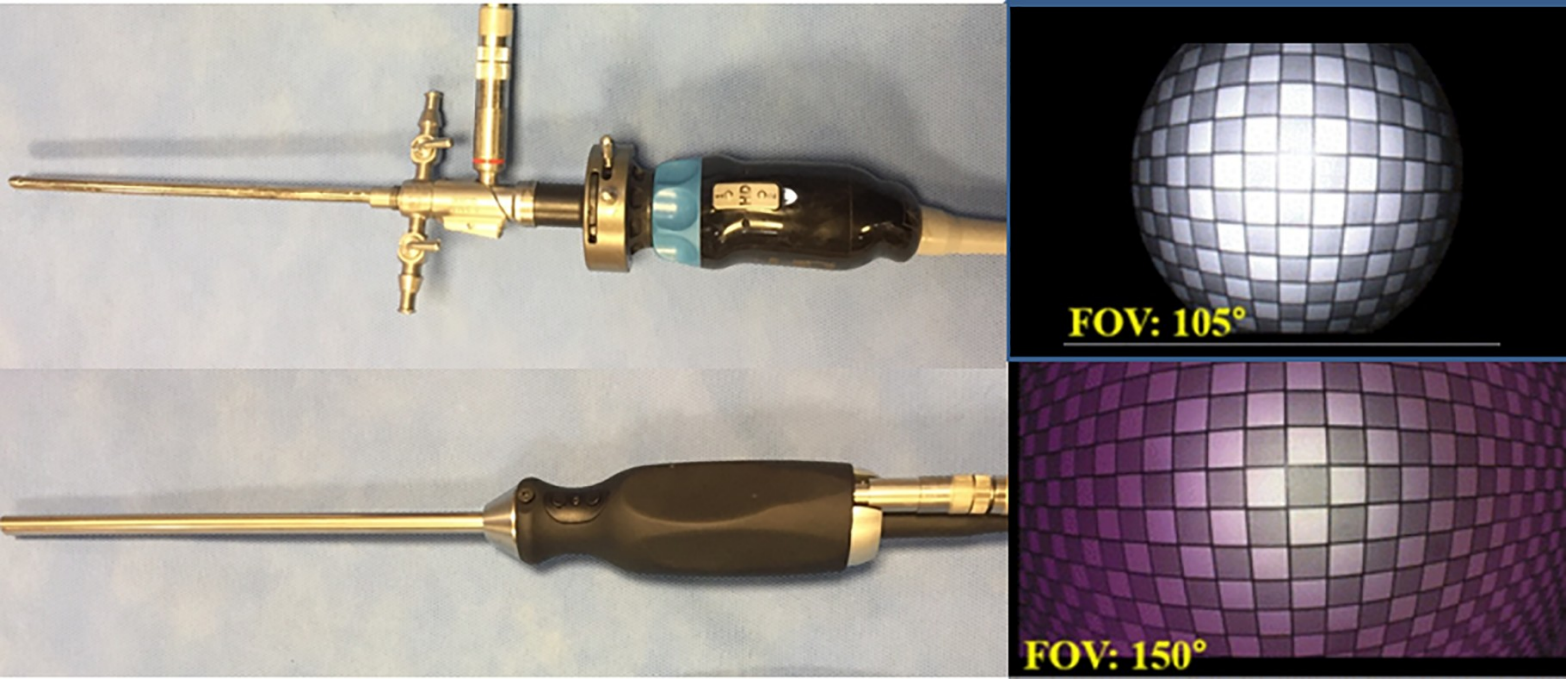
The 2 arthroscopes used in this study, with representative images showing the field of view. (a) The conventional arthroscopic system. (b) The new wide-angle arthroscopic system.

A conventional arthroscopic system (IM4000, IM4120; ConMed Linvatec, Utica, NY, USA) was used to perform the tasks in this study, and compared with a new wide-angle arthroscopic system (MGB Endoscopy Company, Seoul, Korea) that has a high-definition lens with a 150° FOV (Fig 1).

### Arthroscopic tasks

The participants performed a set of tasks using the 2 arthroscopes sequentially in random order. A Latin square counterbalancing technique was used to compensate for learning effects from previous practice [13]. Before the experiment, each participant was given an instructional video manual that covers all the experimental processes and arthroscopic tasks to be performed, as well as provides an introduction to the experimental and surgical instruments. The participants were allowed 10 min to familiarize themselves with the experimental system and surgical instruments. The participants performed the tasks without any assistance.

**Fig 2:**
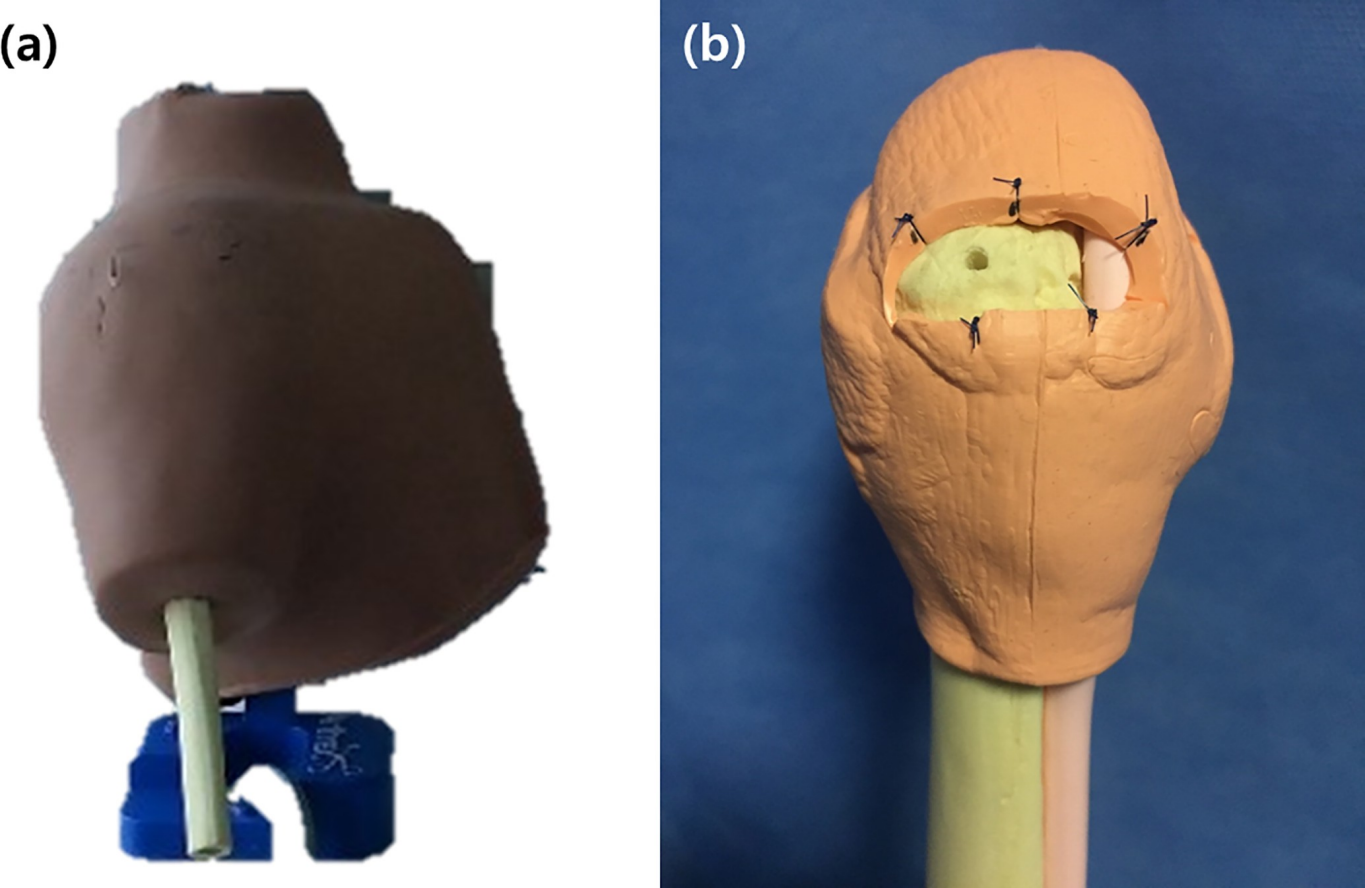
(a) A dry human shoulder model was used for the arthroscopic tasks. (b) Black nylon sutures were made at 5 sites along the lateral border of the rotator cuff (*red arrows*), and the bicipital groove was marked in blue. A pilot hole for an anchor was made at an appropriate position on the greater tuberosity.

A dry right shoulder model (Arthrex, Naples, FL, USA) was used for the tasks (Fig 2A). Black nylon sutures were made at 5 sites along the lateral border of the rotator cuff, and the bicipital groove was marked in blue. A pilot hole for an anchor was made at an appropriate position on the greater tuberosity (Fig 2B). The model and related instruments were set up on a table, with a pair of reflective hand illustrations placed on the table to mark the start and end positions for the participants (Fig 2B).

**Fig 3:**
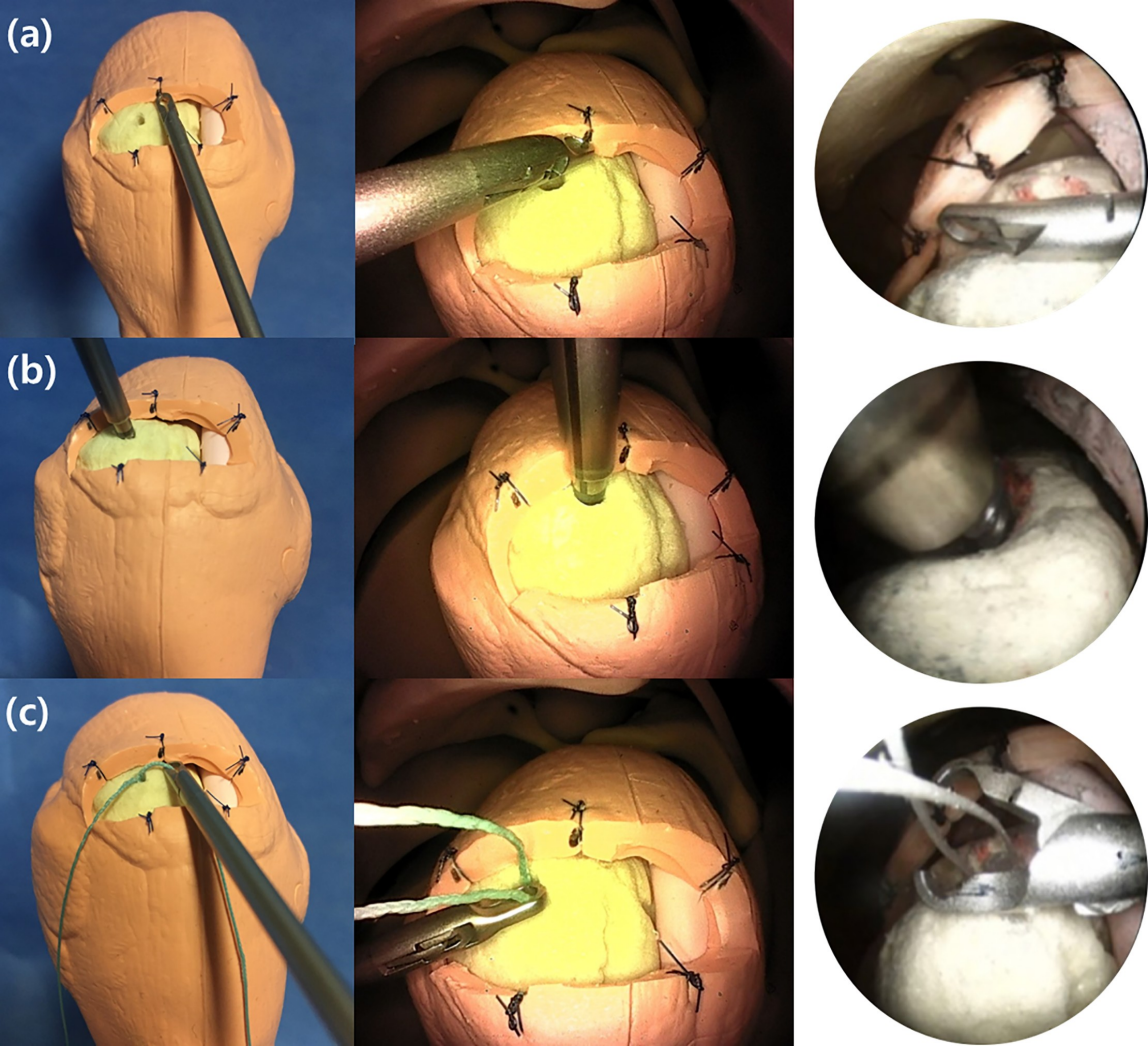
The 3 arthroscopic tasks. (a) Touching 5 points on the rotator cuff twice using a grasper, passing through the anterior portal. (b) Anchor insertion at a predetermined point on the footprint of the rotator cuff. (c) Pulling the suture through the anterior portal by using the grasper.

The 3 assigned tasks, which simulated basic surgical techniques, were as follows: (a) touching the 5 points along the rotator cuff marked by sutures twice using a grasper, passing through the anterior portal; (b) inserting an anchor at the predetermined point on the footprint of the rotator cuff; and (c) pulling the suture through the anterior portal using the grasper (Fig 3A–3C). These 3 tasks have been validated by previous studies [6, 14–16]. Each task was performed 3 times with each scope.

### Hand motion analysis

**Fig 4:**
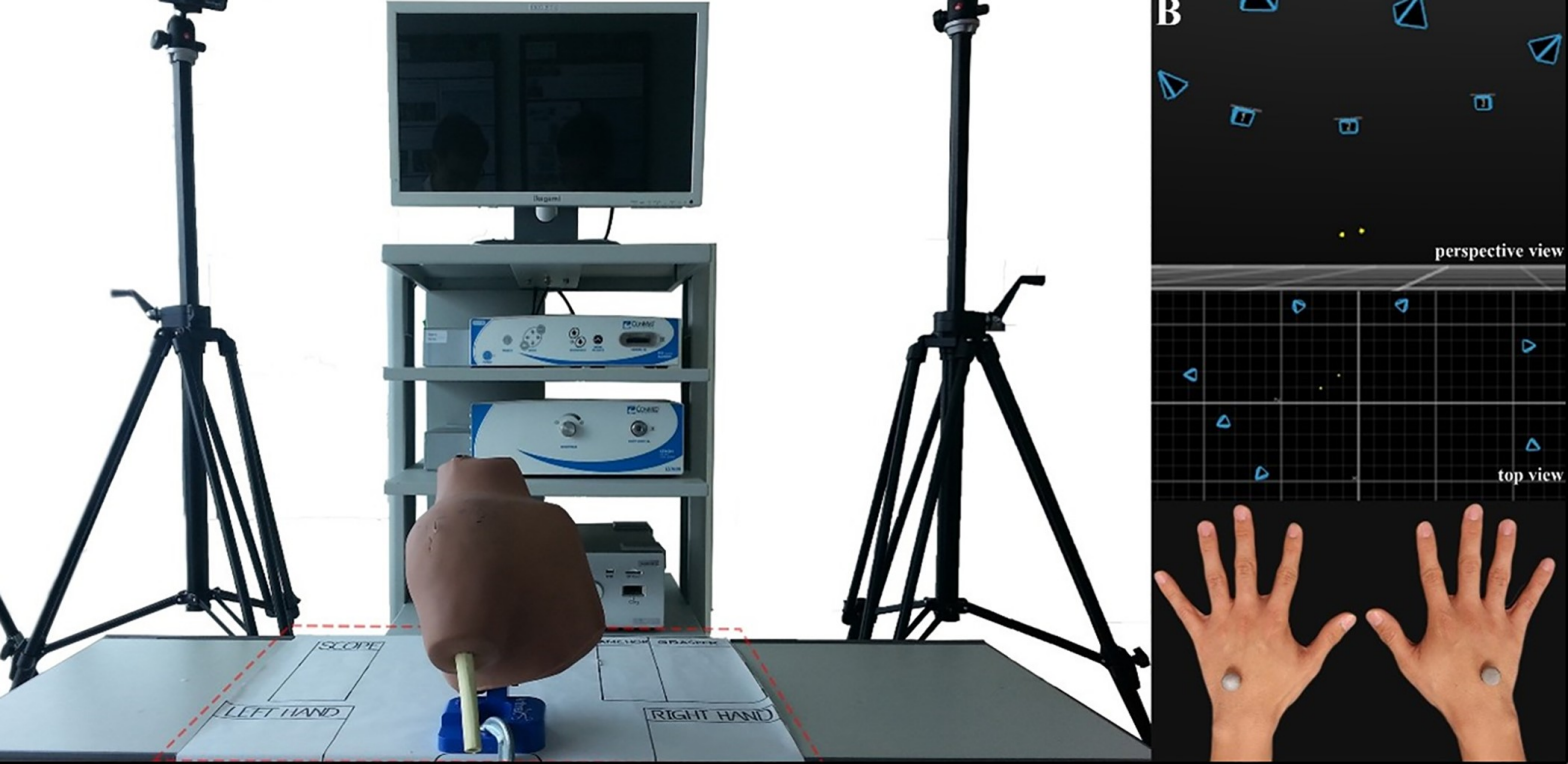
The optical motion analysis system, comprising 8 motion-capture cameras, reflective markers placed on the third metacarpal area of the dorsal side of the participant’s hand to track hand motion, and tracking software.

During the tasks, the participants’ hand movements were tracked using an optical motion analysis system comprising 8 motion-capture cameras (Prime 41; Natural Point Inc., Corvallis, OR, USA) (Fig 4A), 10-mm reflective marker balls placed on the third metacarpal area of the dorsal side of the participants’ hands, and tracking software (Motive: Tracker, Natural Point Inc.) (Fig 4B). The system continuously recorded 3-dimensional data for the 2 reflective markers on each participant’s hands. The following data were collected and analyzed using motion analysis, as described in previous reports [10, 15, 17]: (a) the time taken (in seconds) to complete the tasks, recorded from the time the participant started to lift the hand from the table until the time the task was completed and the participant’s hand was resting on the table; (b) the average acceleration of the hands (m/s^2^); (c) the number of movements made, defined by changes in velocity with respect to the time that exceeded the predetermined threshold value of 10 m/s^2^; and (d) the total path length (m), which was calculated as the total distance traveled by the reflective marker during the task.

By using the collected data, DSJ, a physical index for estimating hand-eye coordination, was calculated in MATLAB 2012b (MathWorks, Torrance, CA, USA) with the following formula:

$$\mathrm{DSJ}=(\int_{t_{1}}^{t_{2}}{x\prime \prime \prime (t)}^{2}dt)*D^{3}/v_{mean}^{2},$$

where D is the duration of the movement and *v_mean_* is the average velocity of the movement.

### Statistical analysis

The time taken to complete the tasks, total path length and number of movements were normally distributed and categorized as continuous variables. DSJ and average acceleration were not normally distributed. An *a priori* power analysis showed that a sample size of 34 attempts of using each arthroscope would have sufficient power to show a significant difference, assuming a significance level of 0.05 and power of 80%. The p-values for the time to complete the tasks, total path length, and number of movements were calculated using a paired t-test, with the Wilcoxon signed rank test used for DSJ and average acceleration. A value of p < 0.05 was considered to indicate statistical significance. The analyses were performed using the SPSS software package (version 20.0; IBM Corp., Armonk, NY, USA).

## Results

DSJ (p = 0.014) and average acceleration (p = 0.039) were the only parameters that showed a statistically significant difference between the 2 arthroscopes. There were no other statistically significant differences between the arthroscopes, including the time taken to complete the tasks (p = 0.282), number of movements (p = 0.323), or total path length (p = 0.142). All other estimated values for the tasks using the 150° arthroscope were lower than those for the tasks using the 105° arthroscope. The collected data are summarized in Table 1.

**Table 1:** Data differences between conventional scope and new wide angled scope.

Values*	105^o^ scope (n = 14)	150^o^ scope (n = 14)	P-value
**Time (s)**	84.14±25.59	89.8±22.51	0.282
**Average acceleration (cm/s2)**	0.15±0.04	0.14±0.04	0.044
**Number of movements**	4724.98±1245.58	4482.64±1109.02	0.323
**Path length (cm)**	5.05±0.91	4.87±0.72	0.142
**DSJ**	16.7±24.52	4.69±7.79	0.008

*Values are mean ± standard deviation

## Discussion

Previous studies have demonstrated that improving the arthroscopist’s view results in better performance; in other words, improved hand-eye coordination leads to better surgical performance [8–10, 17–22]. On the basis of this theory, we introduced the prototype of the wide-angle arthroscope in a previous study [9]. Compared with conventional arthroscopes, which have a 105° FOV, the new arthroscope with a 150° FOV provides unprecedented wide-angle viewing. However, our previous study scientifically demonstrated that the wide-angle view would not simply result in superior performance because various complicated movements are involved; the main issue is identifying a specific tool to estimate movement quality. The widely used global rating scale is an efficient and easy way to estimate surgical performance [23–26]. However, assessment of subjective factors remains to be done because arthroscopy is conducted under the observation of another person [27].

To overcome this subjective limitation, we tried to objectively find a tool for assessing hand-eye coordination, which is highly related to surgical performance. Fortunately, motion tracking systems make it possible to obtain objective data with respect to hand movements [15, 28–31]. By using a motion tracking system, we evaluated the participants’ performance and hand movements according to the time taken to complete the tasks, total path length traveled by the hands, number of movements made, and average acceleration of the hands. From these data, we calculated DSJ, a relatively simple indication of movement quality. A “jerk” is a physical value indicating the rate of change of acceleration for a movement. In DSJ, this is squared to counterbalance any negative values and is presented as a dimensionless parameter to allow it to be comparable. DSJ has previously been used to evaluate several movement disorders [12, 32] and has been considered an effective parameter for quantifying the quality of movement [33, 34]. Hogan and Sternad [12] noted that “a dimensionless jerk-based measure properly quantifies common deviations from smooth, coordinated movement.” This suggests that appropriate measurement of DSJ would reflect the quality of the hand movements of arthroscopists.

The results of our previous study, which demonstrated the validity of DSJ for the objective assessment of hand-eye coordination, indicated that DSJ can be used to objectively assess surgical performance [35]. In the present study, DSJ and average acceleration showed statistically significant differences between the results for the 2 arthroscopes. On the basis of the results, we finally objectively demonstrated the superiority of the new wide-angle arthroscope compared with the conventional arthroscope. we believe that the new wide-angle arthroscope may improve arthroscopic performance and can serve as a superior training tool compared with the conventional arthroscope.

One limitation of this study was the relatively small sample size. Second, measuring the "working" hand using the instrument, the formula will be influenced by the expected increase in motion of the "scope" hand when the scope with the more limited field of view is used. This could be the compounding factor. In addition, the arthroscopic surgical tasks were performed using a dry model, which does not perfectly reflect the clinical situation. To prove the improvement of surgical performance in clinical situation, the 2^nd^ prototype of wide-angle arthroscope is under development and has been used to determine the applicability in cadaveric study. Although some obstacles to apply to surgery have been reported including the torsional image, we believe that the wide-angle arthroscope would give the more benefit than conventional arthroscope in a surgical field. Technical errors and long-term benefits will be also assessed in our future study.

## Conclusion

The surgical performance of novice arthroscopists was improved with the use of the wide-angle arthroscope as compared with the use of the conventional arthroscope.

## Supporting information

S1 File IRB file (IRB for DSJ.pdf). (PDF)

S2 File Data file (data_PONE-D-18-21170.xlsx). (XLSX)
