# Supplementary material for: Improvement of arthroscopic surgical performance using a new wide-angle arthroscope in the surgical training
Source: PLoS One. 2019 Mar 11;14(3):e0203578. doi: 10.1371/journal.pone.0203578 (PMC6411148; doi:10.1371/journal.pone.0203578)
Supplement: S1 File — (PDF) [file pone.0203578.s001.pdf]

주소 : 05505 서울특별시 송파구 올림픽로 43길 88 서울아산병원 TEL : 02-3010-7166, FAX : 02-3010-7318

심의면제 확인서

**과제명**	모의 관절경 수술에서 Dimensionless Squared Jolt(DSJ)를 이용한 손 움직임의 효율성 평가 (면제확인번호 :2017-0292)				
**연구책임자**	**소속** 정형외과	**직위**	교수	**성명**	전인호

**1. 생명윤리 및 안전에 관한 법률에 의거한 인간대상연구 (기본사항이 모두 '예'이고, 아래 항목 중 하나 이상에 해당하는 경우 심의면제가 가능함)**	**예**	**아니오**
**기본사항**		
1) 연구대상자 및 공공에 미치는 위험이 미미한 경우임	☑	☐
2) 일반 대중에게 공개된 정보를 이용하는 연구 또는 개인식별정보를 수집·기록하지 않은 연구	☑	☐
3) 취약한 환경에 있는 연구대상자를 대상으로 하는 연구가 아님	☑	☐
**아래**		
1) 연구대상자를 직접 조작하거나 그 환경을 조작하는 연구 중 다음 어느 하나에 해당하는 연구<br>(1) 약물투여, 혈액채취 등 침습적 행위를 하지 않는 연구<br>(2) 신체적 변화가 따르지 않는 단순 접촉 측정장비 또는 관찰장비만을 사용하는 연구<br>(3) 「식품위생법 시행규칙」 제3조에 따라 판매 등이 허용되는 식품 또는 식품첨가물을 이용하여 맛이나 질을 평가하는 연구<br>(4) 「화장품법」 제8조에 따른 안전기준에 맞는 화장품을 이용하여 사용감 또는 만족도 등을 조사하는 연구	☑	☐
2) 연구대상자와 배아·난자·정자 또는 인체유래물의 기증자를 직접 대면하더라도 연구대상자와 배아·난자·정자 또는 인체유래물의 기증자가 특정되지 않고「개인정보보호법」제23조에 따른 민감정보를 수집하거나 기록하지 않는 연구(사상, 신념, 노동조합, 정당의 가입, 탈퇴, 정치적 견해, 건강, 성생활 등에 관한 정보, 유전자검사 등의 결과로 얻어진 유전정보, 형의 실효 등에 관한 법률 제2조제5호에 따른 범죄경력자료에 해당하는 정보)	☑	☐
3) 연구대상자와 배아·난자·정자 또는 인체유래물의 기증자에 대한 기존의 자료나 문서를 이용하는 연구. 다만, 서울아산병원 의무기록에 직접 접근하는 연구는 제외	☐	☑

**2. 생명윤리 및 안전에 관한 법률에 의거한 인체유래물연구 (기본사항이 모두 '예'이고, 아래 항목 중 하나 이상에 해당하는 경우 심의면제가 가능함)**	**예**	**아니오**
**기본사항**		
1) 인체유래물기증자 및 공공에 미치는 위험이 미미한 경우임	☐	☑
**아래**		

[redacted]

주소 : 05505 서울특별시 송파구 올림픽로 43길 88 서울아산병원 TEL : 02-3010-7166, FAX : 02-3010-7318

1) 연구자가 개인정보를 수집·기록하지 않은 연구 중 다음 어느 하나에 해당하는 연구<br>(1) 인체유래물은행이 수집·보관하고 있는 인체유래물과 그로부터 얻은 유전정보(이하 "인체유래물 등"이라 한다)를 제공받아 사용하는 연구로서 인체유래물 등을 제공한 인체유래물은행을 통하지 않으면 개인정보를 확인할 수 없는 연구<br>(2) 의료기관에서 치료 및 진단을 목적으로 사용하고 남은 인체유래물 등을 이용하여 정확도 검사 등 검사실 정도관리 및 검사법 평가 등을 수행하는 연구<br>(3) 인체유래물을 직접 채취하지 않는 경우로서 일반 대중이 이용할 수 있도록 인체유래물로부터 분리리가공된 연구재료(병원체, 세포주 등을 포함한다)를 사용하는 연구<br>(4) 연구자가 인체유래물 기증자의 개인식별정보를 알 수 없으며, 연구를 통해 얻어진 결과가 기증자 개인의 유전적 특징과 관계가 없는 연구. 다만, 배아줄기세포주를 이용한 연구는 제외	☐	☑
2) 「초·중등교육법」 제2조 및 「고등교육법」 제2조에 따른 학교와 보건복지부장관이 정하는 교육기관에서 통상적인 교육과정의 범위에서 실무와 관련하여 수행하는 연구	☐	☑
3) 공중보건상 긴급한 조치가 필요한 상황에서 국가 또는 지방자치단체가 직접 수행하거나 위탁한 연구	☐	☑

**3. US DHHS 또는 US FDA의 규제를 받는 연구인 경우<br>(아래 6가지 범주 중 하나 이상에 해당되어야 하고, 해당되는 범주의 세부 내용이 모두 '예'이어야 함)**		**예**	**아니오**
범주 1	1) 일반적으로 받아들여지는 교육환경에서 수행되는 연구임	☐	☑
	2) 일반적인 교육과정을 포함함(예, 정규 및 특수 교수법 전략에 대한 연구<br>교수법, 교과과정 또는 수업관리 방법들 간의 비교 혹은 효과성에 대한 연구)	☐	☑
	3) 연구대상자에 수감자를 포함하지 않은 연구	☐	☑
	4) U.S. FDA 규제를 받지 않은 연구	☐	☑
범주 2	1) 교육용 시험(인지, 진단, 적성, 성취도), 설문조사, 면접조사, 대중 행동 관찰 등이 한가지 이상 포함된 연구	☐	☑
	2) 연구대상자의 정보가 적절하게 보호되지 않아서 연구대상자가 민형사상의 책임을 지게 되거나 재정적인 상황, 고용, 보험 또는 명성을 악화시키거나 오명을 씌우게 하는 경우가 없음	☐	☑
	3) 연구대상자를 식별하거나 직·간접적인 식별자를 통해 연구대상자를 식별할 수 있는 상태로 정보가 기록되지 않음	☐	☑
범주 3	1) 교육용 시험(인지, 진단, 적성, 성취도), 설문조사, 면접조사, 대중 행동 관찰 등이 한가지 이상 포함된 연구	☐	☑
	2) 다음 조건 중 어느 하나에 해당될 때<br>· 공직에 근무 중이거나 근무 예정인 자를 대상으로 하는 연구 또는 만 12세 미만의 자를 포함하지 않는 연구<br>· 연구대상자의 사적 정보에 대한 비밀이 법률에 따라 연구기간 동안 또는 영구적으로 보장되는 경우	☐	☑
	3) 연구대상자에 수감자를 포함하지 않은 연구	☐	☑
	4) U.S. FDA 규제를 받지 않은 연구	☐	☑
범주 4	1) 문서, 기록, 검사결과, 문헌 그리고 병리적 표본 또는 진단표본 등과 같이 기존에 확보된 자료를 대상으로 하는 연구	☐	☑
	2) 다음 조건 중 어느 하나에 해당될 때<br>· 공개적으로 이용 가능한 자료<br>· 직접적으로 또는 연구대상자와 연결된 식별자를 통해 연구대상자를 확인할 수 없는 연구	☐	☑
범주 5	1) 프로젝트가 연구이거나 시범사업임	☐	☑

[redacted]

주소 : 05505 서울특별시 송파구 올림픽로 43길 88 서울아산병원 TEL : 02-3010-7166, FAX : 02-3010-7318

범주 5	2) 연방정부나 기관장의 승인 하에 수행되는 연구	☐	☑
	3) 다음과 같은 항목을 평가하기 위한 연구<br>· 공익 또는 서비스 프로그램<br>· 이러한 프로그램 하에 얻어지는 이익 또는 서비스의 절차<br>· 이러한 프로그램 또는 절차의 변경 또는 대체 가능성<br>· 이러한 프로그램 하에 이익 또는 서비스를 위한 지불수준 또는 방법의 변경 가능성	☐	☑
	4) 연구가 공공의 이익이나 서비스로 이어질 때	☐	☑
	5) 연구가 특수한 연방정보의 권한으로 실시될 때	☐	☑
	6) IRB 심의 요구조건이 없을 때	☐	☑
	7) 비침습적 또는 연구대상자의 사생활 침해가 포함되지 않은 연구	☐	☑
	8) 연구대상자에 수감자를 포함하지 않은 연구	☐	☑
	9) U.S. FDA 규제를 받지 않은 연구	☐	☑
범주 6	1) 식품의 질과 맛 평가 또는 소비자 선호도 확인을 위한 연구	☐	☑
	2) 다음 조건 중 어느 하나에 해당될 때<br>· 식품첨가물이 없거나<br>· 정부기관에서 안전성을 승인한 식품첨가물만을 사용한 식품 대상	☐	☑
	3) 연구대상자에 수감자를 포함하지 않은 연구	☐	☑

최종결과	심의면제 가능여부 판정일	2017년 03월 08일
	본 신규과제는 IRB에서 규정한 심의면제 조건에 부합하여 심의를 면제합니다. 판정일 이후부터 연구개시가 가능하며 본 확인서를 보관하시기 바랍니다.	

기타 의견

본 위원회에서는 연구자가 제출하신 심의면제를 검토한 결과 승인하기로 결정하였습니다. 심의면제 연구라도 AMC IRB SOP와 HRPP 규정을 준수하여야 함을 유의하시고 연구를 진행하여 주시기 바랍니다.

제출자료 목록 및 버전번호

연구계획서(1.0)

증례기록서(1.0)

[redacted]

주소 : 05505 서울특별시 송파구 올림픽로 43길 88 서울아산병원 TEL : 02-3010-7166, FAX : 02-3010-7318

임상연구심의위원회/기관생명윤리위원회	위원장 이무송 (인)

본 임상연구심의위원회는 국제표준화추진회의(ICH), 의약품임상시험관리기준/의료기기임상시험실시기준(KGCP) 및 생명윤리및안전에관한법률 등 관련 법규를 준수합니다. 본 연구와 이해상충관계가 있는 위원이 있을 경우 해당 위원은 연구의 심의에서 배제하였습니다.

ASAN MEDICAL CENTER 서울아산병원

[redacted]
